# Lanthanide (Eu^3+^/Tb^3+^)-Loaded γ-Cyclodextrin Nano-Aggregates for Smart Sensing of the Anticancer Drug Irinotecan

**DOI:** 10.3390/ijms23126597

**Published:** 2022-06-13

**Authors:** Yaowei Guo, Jin Liu, Qinglin Tang, Cuicui Li, Yanying Zhang, Yao Wang, Yanxin Wang, Yupeng Bi, Christopher D. Snow, Matt J. Kipper, Laurence A. Belfiore, Jianguo Tang

**Affiliations:** 1 Institute of Hybrid Materials, National Center of International Joint Research for Hybrid Materials Technology, National Base of International Sci. & Tech. Cooperation on Hybrid Materials, Qingdao University, 308 Ningxia Road, Qingdao 266071, China; yw1996guo@163.com (Y.G.); liujin0620@126.com (J.L.); a15666920912@163.com (Q.T.); 17853482427@163.com (C.L.); zyyaaaa@163.com (Y.Z.); wangyaoqdu@126.com (Y.W.); wangyanxin@qdu.edu.cn (Y.W.); b15254156675@163.com (Y.B.); 2 Department of Chemical and Biological Engineering, Colorado State University, Fort Collins, CO 80523, USA; christopher.snow@colostate.edu (C.D.S.); belfiore@engr.colostate.edu (L.A.B.)

**Keywords:** lanthanide (Eu^3+^, Tb^3+^) complex, γ-cyclodextrin, nano-aggregate, sensing, anticancer drug, irinotecan

## Abstract

The clinical use of anticancer drugs necessitates new technologies for their safe, sensitive, and selective detection. In this article, lanthanide (Eu^3+^ and Tb^3+^)-loaded γ-cyclodextrin nano-aggregates (ECA and TCA) are reported, which sensitively detects the anticancer drug irinotecan by fluorescence intensity changes. Fluorescent lanthanide (Eu^3+^ and Tb^3+^) complexes exhibit high fluorescence intensity, narrow and distinct emission bands, long fluorescence lifetime, and insensitivity to photobleaching. However, these lanthanide (Eu^3+^ and Tb^3+^) complexes are essentially hydrophobic, toxic, and non-biocompatible. Lanthanide (Eu^3+^ and Tb^3+^) complexes were loaded into naturally hydrophilic γ-cyclodextrin to form fluorescent nano-aggregates. The biological nontoxicity and cytocompatibility of ECA and TCA fluorescent nanoparticles were demonstrated by cytotoxicity experiments. The ECA and TCA fluorescence nanosensors can detect irinotecan selectively and sensitively through the change of fluorescence intensity, with detection limits of 6.80 μM and 2.89 μM, respectively. ECA can safely detect irinotecan in the cellular environment, while TCA can detect irinotecan intracellularly and is suitable for cell labeling.

## 1. Introduction

The drugs used in current clinical practice have both beneficial therapeutic effects and undesirable side effects [1]. This is especially true for many anticancer drugs [2]. Anticancer drugs generally have a narrow therapeutic index [3], which means that the toxic dose that produces side effects is very close to the dose that exerts antitumor activity [4,5]. Minimizing side effects while maximizing therapeutic effects is an important goal [6,7] that can be best achieved if the drug concentration can be maintained at an optimized value [8,9,10]. Various analytical techniques have been used to detect and quantify the blood concentration levels of anticancer drugs. Among these analytical techniques, electrochemical methods, radioimmunoassays, immunohistochemistry, gas chromatography, and high-performance liquid chromatography can all detect specific anticancer drugs [11,12,13,14]. However, these methods have limitations, such as high equipment cost and complexity of operation. Moreover, they may not provide a rapid or direct measure of the drug concentration at the site of a tumor. Since most anticancer drugs kill cancer cells, they also have toxicity toward normal tissues and organs, particularly those containing cells that rapidly divide, such as bone marrow and the gastrointestinal tract [6,15]. Spatially resolved, real-time, safe, sensitive, and selective detection of anticancer drugs could enable local monitoring of drug concentration, improving the personal tuning of drug treatments.

Irinotecan (CPT-11) is a semi-synthetic water-soluble derivative of camptothecin a topoisomerase ⅰ inhibitor, which plays a key role in cancer treatment by interfering with DNA replication and inducing apoptotic cell death [6,16]. Its chemical structure is shown in Figure S1 [17]. It also shows obvious dose-limiting toxicity, such as delayed diarrhea and bone marrow suppression, which greatly limits the treatment window [18,19]. CPT-11 is an excellent candidate drug for which to develop a drug sensor, because it has a narrow therapeutic index and because it exhibits wide inter-individual differences in pharmacokinetic and pharmacodynamic behavior [20,21]. A variety of methods to detect CPT-11 have been reported, including electrochemical analysis [17,22], high-performance liquid chromatography coupled to tandem mass spectrometry [23,24], and reversed-phase high-performance coupled to liquid chromatography coupled to UV detection [25]. Although these methods can analyze and detect CPT-11, they have complex operations, long assay times, and the measurement requires a centralized and well-equipped laboratory and skilled operators. In addition, they are not suitable for the control and personalization of drug dosage based on therapeutic drug detection [26]. A sensitive, easy-to-use, cost-effective detection method is required to achieve the ideal dosage amount and dosage time, avoiding toxic effects while maintaining efficacy.

In the development of sensors or probe systems, rare earth hybrid materials are widely used due to their sensitivity and excellent fluorescent properties [27,28]. In situ sensing in complex biological environments requires higher stability, better sensitivity, narrow emission, long fluorescence lifetime, and good biocompatibility [29,30]. Lanthanide ions have narrow emission bands, large stokes shifts, long fluorescence lifetimes, and high quantum yields [31,32]. However, due to the prohibition of the 4f-4f transition, the absorption and emission intensity of the rare earth ion itself is very weak [33]. When lanthanide ions are coordinated with small molecular organic ligands (such as 2-thienyl trifluoroacetone (TTA), acetylacetone (acac), and 1,10-phenanthroline (phen)), the fluorescent properties of lanthanide complexes can be significantly improved through the antenna effect [34,35,36]. Therefore, if the local environment of the coordination complex changes, such as via ligand exchange, these changes can be sensitively detected by the resulting change in fluorescence intensity. However, the coordination with organic ligands makes the resulting lanthanide element complexes hydrophobic and incompatible with the biological environment [37,38]. If lanthanide complexes are to be widely used in biological applications, they must be compatibilized with aqueous environments.

Cyclodextrins (CDs) are cyclic oligomers composed of a series of D-glucopyranose units connected by α-1,4 glycosidic bonds, usually consisting of 6–12 glucose units [39]. The three most common cyclodextrins are α-, β-, and γ-cyclodextrins (α-, β-, and γ-CD), which are composed of 6, 7, and 8 glucose units, respectively [40,41]. Among them, the γ-CD cavity has the largest volume, with a diameter of 0.95 nm and a cavity volume of 427 Å^3^ [41,42]. Its chemical structure is shown in Figure S2 [43]. Cyclodextrin molecules have a hydrophobic inner cavity, while the outer side and the port are hydrophilic [44,45,46]. Cyclodextrin inclusion complexes are compounds formed by one or more suitable guest molecules that enter the cavity of cyclodextrin in whole or in part through non-covalent bonds (such as hydrophobic interactions) [47,48]. The hydrophobic cavity of cyclodextrin has the ability to include small molecules, oligonucleotides, proteins, and other compounds [49,50,51].

**Figure 1:**
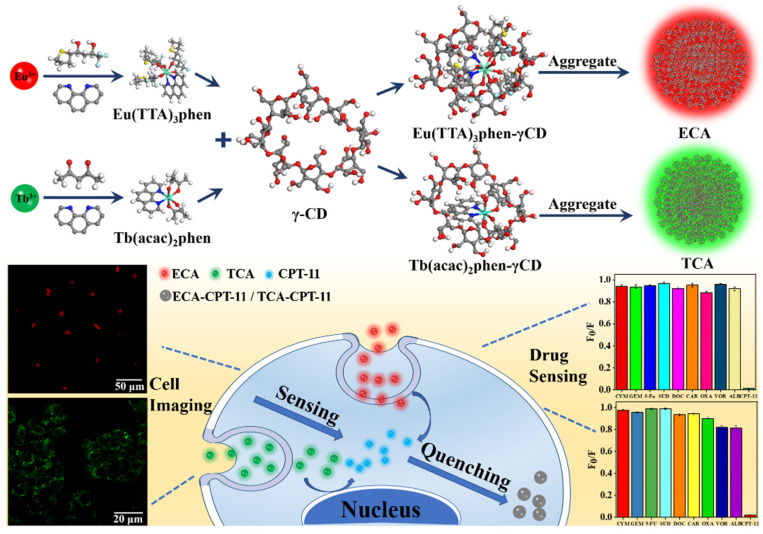
Schematic illustration showing γ-CD encapsulation of Eu^3+^/Tb^3+^ complexes and selective detection of CPT-11.

The lanthanide complexes we have prepared have low polarity and poor water solubility, but they meet the volume requirement for entering the γ-CD cavity. Inside the CD cavity, water forms a high-energy clathrate, which can be displaced by a lanthanide complex. As shown in Figure 1, we used Materials Studio to construct the three-dimensional structural model of Eu^3+^/Tb^3+^ complex and γ-CD, and described the inclusion of γ-CD molecule to Eu^3+^/Tb^3+^ complex molecule. The inclusion of Eu^3+^/Tb^3+^ complex by γ-CD further generated secondary agglomeration to form ECA/TCA nano-aggregates.

In this work, we used a self-assembly strategy to obtain lanthanide (Eu^3+^ and Tb^3+^)-loaded γ-cyclodextrin nano-aggregates. Including the lanthanide complexes in the γ-CD cavity imparts water solubility and cytocompatibility, resulting in low-cost and high-fluorescence drug-sensing ECA and TCA nano-aggregates. Moreover, in the presence of CPT-11, the fluorescent Eu^3+^ and Tb^3+^ complex and the drug are co-located so that the drug can be sensed based on the co-location in the same nanostructure. The ECA and TCA nano-aggregates exhibit fast sensing performance, low detection limit, high sensitivity, and selective identification of CPT-11 in an aqueous medium (Figure 1). In addition, cell interaction experiments show that the ECA and TCA have good cell compatibility and lack toxicity. Therefore, ECA and TCA can be used for biocompatible and water-soluble drug sensing with in-situ drug concentration sensing capabilities.

## 2. Experiments

### 2.1. Materials

Europium chloride hexahydrate (EuCl_3_·6H_2_O) and terbium chloride hexahydrate (TbCl_3_·6H_2_O) were purchased from Shandong Desheng Rare Earth Co., Ltd (Shandong, China). 2-Thienyl trifluoroacetone (TTA), acetylacetone (acac), 1,10-phenanthroline (anhydrous) (phen), and γ-cyclodextrin (γ-CD) were purchased from Shanghai Maclin Biochemistry Co., Ltd (Shanghai, China). Irinotecan (CPT-11), cyclophosphamide monohydrate (CYM), gemcitabine (GEM), 5-fluorouracil (5-FU), sulfadiazine (SUD), dodecaxel (DOC), carvanol (CAR), oxaliplatin (OXA), Vorinotat (VOR), and albendazole (ALB) were purchased from Aladdin Industries Corporation (Shanghai, China). Other chemical reagents were acquired from Shanghai Chemical Reagents Co., Ltd (Shanghai, China). Deionized water was used for preparing all aqueous solutions. All of the above products are of analytical grade and were used without further purification.

### 2.2. Preparation of Lanthanide (Eu^3+^ and Tb^3+^)-Loaded γ-Cyclodextrin Nano-Aggregates

Eu^3+^ and Tb^3+^ complexes were synthesized according to methods described in publications previously reported by us [31]. EuCl_3_·6H_2_O, TbCl_3_·6H_2_O, TTA, acac, and phen were dissolved in anhydrous ethanol and γ-CD was dissolved in deionized water. The EuCl_3_·6H_2_O and TTA were mixed according to the concentrations shown in Table 1 and stirred for 30 min at room temperature. Then the pH of the mixture was adjusted to 7–8, using 1 mol/L NH_3_·H_2_O. After neutralization, phen was added to the solution in the amounts listed in Table 1, and the solution was constantly stirred for 2 h. The obtained Eu^3+^ complex was colorless, transparent, and soluble in ethanol. Finally, the γ-CD solution was added to the solution and stirred for 1 h to form the γ-CD inclusion compound (ECA). Similarly, TbCl_3_·6H_2_O and acac were also mixed according to the concentrations shown in Table 1, and stirred at room temperature for 30 min. The pH of the mixture was adjusted to 7–8, using 1 mol/L NH_3_·H2O solution. Then phen was added to the solution in the amounts listed in Table 1, and the solution was constantly stirred for 2 h. The resulting Tb^3+^ complex is colorless, transparent, and soluble in ethanol. Finally, the γ-CD solution was added and stirred for 1 h to form a γ-CD inclusion compound (TCA).

### 2.3. Fluorescence Properties of Lanthanide (Eu^3+^ and Tb^3+^)-Loaded γ-Cyclodextrin Nano-Aggregates

The fluorescence excitation and emission spectra of the ECA and TCA were measured. For these experiments, the excitation wavelength of Eu^3+^ was 382 nm and the emission wavelength was set to 612 nm, the emission slit width was 0.5 nm. For Tb^3+^, the excitation was performed at 334 nm, the emission wavelength was 546 nm, and the emission slit width was 1 nm. The fluorescence lifetime and quantum yield of the samples were also measured.

### 2.4. ECA and TCA Smart Sensing of Anticancer Drugs

#### 2.4.1. Detection of Irinotecan (CPT-11)

CPT-11 solutions were prepared with concentrations of 1 μm to 1 mM. From these CPT-11 solutions, 1 mL was added to 3 mL of 1 mM ECA and 3 mL of 10 mM TCA solutions and stirred for 0.5 h at room temperature in the dark. Then, the obtained samples were subjected to fluorescence spectrophotometry. The excitation wavelength for Eu^3+^ was 382 nm, the emission wavelength was 612 nm, and the emission slit width was 0.5 nm. The excitation wavelength of Tb^3+^ was 334 nm, the emission wavelength was 546 nm, and the emission slit width was 1 nm. The limit of detection (LOD) of CPT-11 was calculated for both ECA and TCA. All the samples were tested in triplicate (*n* = 3) with identical parameters at room temperature.

#### 2.4.2. Sensitivity of ECA and TCA to Other Anticancer Drugs

Stock solutions (1 mM) of ten drugs (CPT-11, CYM, GEM, 5-FU, SUD, DOC, CAR, OXA, VOR, and ALB) were prepared. Then, 1 mL of 1 mM solutions of different anticancer drugs were added to 3 mL of 1 mM ECA and 3 mL of 10 mM TCA solutions, respectively, and 800 rpm stirring was continued for 0.5 h at room temperature. Spectrophotometry was performed as described above.

### 2.5. Cytocompatibility Measurement

The cytotoxicity of ECA and TCA was determined using HepG2 cells (human hepatoma cells) using a standard MTT assay protocol. HepG2 cells in the logarithmic growth phase were added to wells of a 96-well plate accordingly at 1 × 10^4^/well, and the cells were incubated (5% CO_2_ at 37 °C) until the cells adhered to the wells. Cells were then treated with either a control (Eu(TTA)_3_phen or Tb(acac)_2_phen lanthanide complexes or γ-CD), or experimental (ECA at 1, 2, 4, 8, and 16 mM or TCA at 2.5, 5, 10, 20, and 40 mM) treatment. Cells were then incubated for an additional 24 h. After 24 h, the medium containing the experimental or control treatments was removed. Cells were washed with cell culture medium three times, and 100 μL of medium containing 0.5 mg/mL of the MTT reagent was added to each well. Then the cells were incubated (5% CO_2_, 37 °C) for 4 h. The supernatant was removed and replaced with 100 μL of DMSO in each well. After gently shaking for 10 min, the absorbance at 570 nm was measured. Five biological replicates (*n =* 5) were used for each condition.

### 2.6. Cell Imaging

Human hepatoma HepG2 cells were imaged using confocal laser scanning microscopy. HepG2 cells were incubated in the cell culture medium for 24 h. A 2 mM ECA solution and a 20 mM TCA solution were mixed with the medium 1:1 and then added to the cells for 1 h. After removing the supernatant, the cells were washed with cell culture medium, and 2 mL of cell culture medium was used for imaging by laser scanning confocal microscopy. HepG2 cells with ECA and HepG2 cells with TCA were incubated with 100 μL of 100 μM CPT-11 for 30 min, respectively. After removal of the CPT-11 solution, the samples were washed, and 2 mL of cell culture medium was added for imaging by laser scanning confocal microscopy.

### 2.7. Characterization of ECA and TCA

The morphology and elemental mapping of ECA and TCA were characterized by transmission electronic microscopy (TEM) with a JEM-2100F (JEOL Ltd., Tokyo, Japan). Each sample was ultrasonicated for 5–6 min. The sonicated solution was then dropped onto a copper grid and the solvent was evaporated at room temperature for 1 or 2 s. The particle size of ECA and TCA was determined by dynamic light scattering (DLS) using a Malvern laser particle size analyzer (Malvern Zetasizer Nano ZS90, Shanghai, China). Each sample was ultrasonicated for 5–6 min before testing. The photoluminescence spectra of the samples were obtained by a photoluminescence spectrometer (FLS1000, Edinburgh, UK) and the fluorescence lifetime and quantum yield of the ECA and TCA were evaluated. Atomic force microscopy (AFM) and photo-induced force microscopy (PiFM) were performed on a PiFM instrument (Molecular Vista, California, U.S.). X-ray photoelectron spectroscopy (XPS) was performed using a Thermo Scientific K-Alpha instrument. Cell imaging was performed using a laser scanning confocal microscope (ZEISS, model: LSM880, Oberkochen, Germany).

## 3. Results and Discussion

### 3.1. Lanthanide (Eu^3+^/Tb^3+^)-Loaded γ-Cyclodextrin Nano-Aggregates

**Figure 2:**
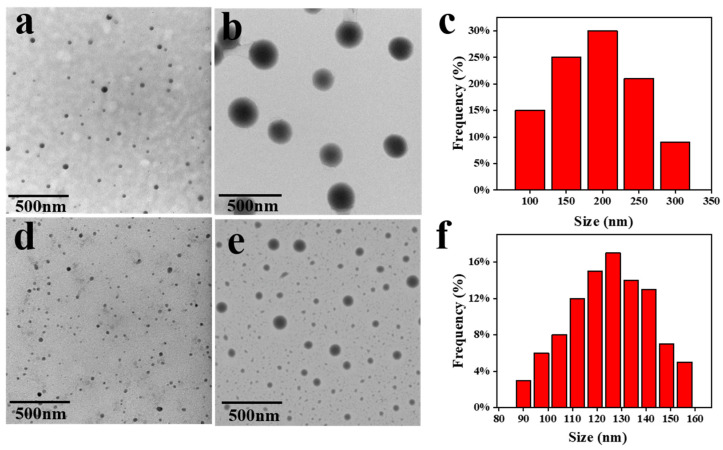
TEM images of ECA and TCA samples prepared according to Table 1. (**a**) Sample A, (**b**) sample B, (**d**) sample C, (**e**) sample D. (**c**) Size distribution of sample B by DLS and (**f**) Size distribution of sample D by DLS.

The ability of γ-cyclodextrin to serve as a host for hydrophobic guest molecules and complexes provides the opportunity to prepare self-assembled nano-aggregates into which lanthanide complexes (Eu(TTA)_3_phen or Tb(acac)_2_phen) can be loaded. Transmission electron microscopy images and DLS data were used to determine the size and morphology, as shown in Figure 2. The sample indices A, B, C, and D refer to the annotations in Table 1. Figure 2a,b is TEM of nano-aggregates prepared from γ-CD loaded with different concentrations of Eu^3+^ complexes. Due to the different concentrations of ligand and Eu^3+^, these samples exhibited spherical nanoparticles of varying sizes. In the low concentration sample A (Figure 2a), the size of spherical nanoparticles was observed to be below 100 nm, while sample B (Figure 2b) showed increased spherical nanoparticle size (100 nm to 300 nm) due to an increase of the Eu^3+^ complex concentration. Similarly, the TCA nanoparticles had a spherical morphology at both concentrations. In the low concentration sample C (Figure 2d), the size of spherical nanoparticles was observed to be below 50 nm. Finally, the size range of sample D (Figure 2e) was 90–160nm. The size distributions obtained via DLS for ECA Sample B, and TCA sample D are shown in Figure 2c and Figure 2f, respectively. ECA had a higher tendency to agglomerate than TCA, so the size of the resulting nanoparticles was larger. Considering the size, stability, and fluorescence intensity of the sample, Eu^3+^ and Tb^3+^ concentrations in sample B and sample D were chosen for further experiments. Notably, nanoparticles in this size range meet the requirements of the enhanced permeability and retention (EPR) effect, whereby nanoparticles may avoid hepatic and splenic filtration, and accumulate in tumor tissues [52]. In addition, spherical nanoparticles have a faster internalization rate and are more easily absorbed by cells [53].

**Figure 3:**
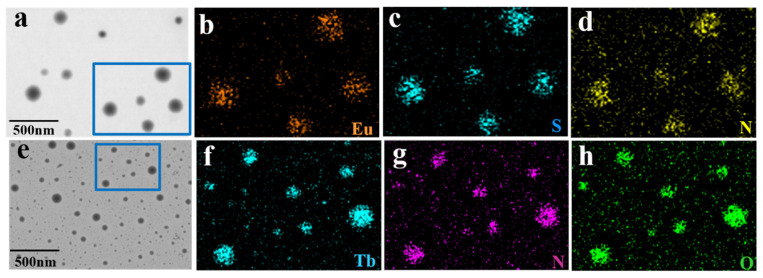
(**a**) High-resolution TEM of sample B, (**b**) elemental mapping of the distribution of Eu, (**c**) elemental mapping of the distribution of S, (**d**) elemental mapping of the distribution of N. (**e**) High-resolution TEM of sample D, (**f**) elemental mapping of the distribution of Tb, (**g**) elemental mapping of the distribution of N, (**h**) elemental mapping of the distribution of O.

To further explore the structures of ECA and TCA, and to evaluate the elemental distribution of spherical nanoparticles, high-resolution TEM was performed. Figure 3 shows the distribution of Eu, Tb, S, N, O, elements from sample B and sample D, and Figure S3 shows the relative content of the corresponding elements. Figure 3a and Figure 3e show the TEM images of ECA and TCA, respectively. The distributions of Eu (orange), S (blue), and N (yellow) in the ECA spherical nanoparticles are shown in Figure 3b–d. Elemental mapping of ECA revealed a uniform distribution of Eu on the spherical nanoparticles (Figure 3b). The S and N elements, present in the TTA and phen ligands, were distributed throughout the nanoparticles, as shown in Figure 3c and Figure 3d, respectively. The distributions of Tb (blue), N (purple), and O (green) in TCA spherical nanoparticles are shown in Figure 3f–h. The EDS elemental mapping by TCA revealed the distribution of the Tb element in spherical nanoparticles. The distribution of N element was similar to that of the Tb element, and the N element only existed in the ligand phen, which proved the complexation between the ligand and terbium. Therefore, the formation of ECA and TCA nanoparticles can be seen from the elemental mapping images.

In addition, the complexation of Eu^3+^ and Tb^3+^ complexes and the formation of ECA/TCA were investigated by X-ray photoelectron spectroscopy. Figures S4a and S4e show the XPS survey spectra of Eu^3+^ complex and Tb^3+^ complex, respectively. Figure S4b shows the XPS spectrum of Eu3d. The binding energy peaks of Eu3d3 and Eu3d5 appeared at 1163.78 eV and 1134.38 eV, respectively, and increased by 8.78 eV and 8.38 eV, respectively, compared with the standard binding energy peaks 1155 eV and 1126 eV of Eu3d3 and Eu3d5, confirming the existence of Eu^3+^ in the sample and its complexation with the ligand. Figure S4c shows the N1s peak of Eu^3+^ complex at 399.04 eV compared with the standard N1s peak of 398.4 eV, indicating that N in ligand phen was successfully coordinated. Figure S4d shows that the XPS spectrum of O1s has two peaks corresponding to C–O (531.38 eV) and C=O (530.68 eV), respectively. Compared with the standard binding energies of 533.0 eV and 532.0 eV of C–O and C=O, the binding energies of C–O and C=O in the complex decreased by 1.62 eV and 1.32 eV, respectively, which indicated that O in the ligand TTA was coordinated with Eu^3+^ to form a complex. Similarly, Figure S3f shows the XPS spectrum of Tb3d corresponding to two peaks of Tb3d3 (1276.48 eV) and Tb3d5 (1242.38 eV), respectively. Figure S3g shows the peak of N1s in the ligand Phen (398.99 eV). Figure S3h shows the XPS spectra of O1s corresponding to C–O (531.78 eV) and C=O (531.18 eV), respectively. Among these peaks, the position of the peak changed relative to the standard binding energy, confirming the formation of the complexes.

### 3.2. Photophysical Properties of Lanthanide (Eu^3+^ and Tb^3+^)-Loaded γ-Cyclodextrin Nano-Aggregates

**Figure 4:**
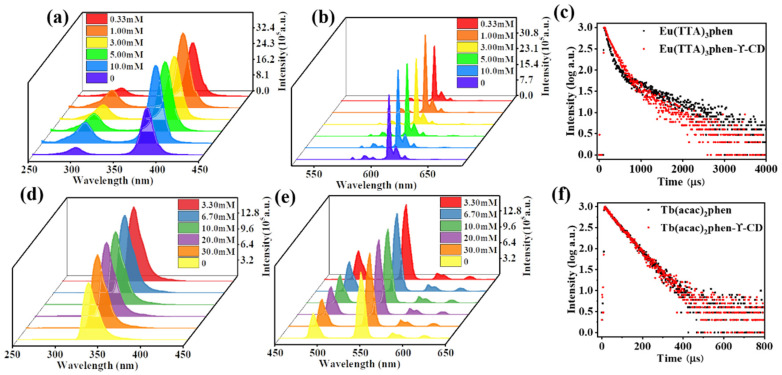
Excitation spectra of (**a**) Eu^3+^ and (**d**) Tb^3+^ complexes with and without γ-cyclodextrin encapsulation. Emission spectra of (**b**) Eu^3+^ and (**e**) Tb^3+^ complexes with and without cyclodextrin encapsulation. The fluorescence lifetime of (**c**) Eu^3+^ and (**f**) Tb^3+^ complexes with and without cyclodextrin encapsulation.

The photophysical properties of ECA and TCA were measured by fluorescence spectrophotometry. Figure 4a,b shows the fluorescence excitation and emission spectra of Eu^3+^ complexes and ECA formed using different concentrations of γ-CD. The Eu^3+^ complex had a wider absorption band at 360–420 nm, with the maximum excitation wavelength at 382 nm. In Figure 4b, after adding γ-CD, the emission intensity of Eu^3+^ at the strongest emission wavelength of 612 nm increased significantly. Keeping the Eu^3+^ ion concentration unchanged and changing the γ-CD concentration, the fluorescence emission intensity was the largest when the Eu^3+^ complex and γ-CD concentration ratio was 1:3. There are five characteristic absorption peaks in the emission spectra of all complexes in Figure 4b, corresponding to ^5^D_0_-^7^F_0_ (580 nm), ^5^D_0_-^7^F_1_ (590 nm), ^5^D_0_-^7^F_2_ (612 nm), ^5^D_0_-^7^F_3_ (652 nm), ^5^D_0_-^7^F_4_ (704 nm) transitions of Eu^3+^. Figure 4d,e shows the fluorescence excitation and emission spectra of Tb^3+^ complexes and TCA formed under different concentrations of γ-CD. It can be seen from Figure 4d that the Tb^3+^ complex had a broad absorption band between 325 and 400 nm. This broad band is caused by the π–π* electronic transition between acac and phen in the complex. The Tb^3+^ complex had a maximum excitation wavelength of 334 nm. In Figure 4e, after adding γ-CD, the emission intensity of Tb^3+^ at the strongest emission wavelength of 546 nm increases significantly. Keeping the Tb^3+^ ion concentration unchanged and changing the γ-CD concentration, the fluorescence emission intensity was the largest when the concentration ratio of Tb^3+^ complex to γ-CD was 1:1. The emission spectra of all complexes in Figure 4e show four characteristic peaks corresponding to the ^5^D_4_-^7^F_6_ (490 nm), ^5^D_4_-^7^F_5_ (546 nm), ^5^D_4_-^7^F_4_ (580 nm), and ^5^D_4_-^7^F_3_ (620 nm) transitions of Tb^3+^. Compared with Eu(TTA)_3_phen and Tb(acac)_2_phen complexes, the fluorescence intensity of the strongest emission bands of ECA and TCA were significantly enhanced at 612 nm (Eu^3+^) or 546 nm (Tb^3+^). This enhancement arises because the hydrophobic cavity of γ-CD helps to prevent quenching of the Eu^3+^ and Tb^3+^ complex in solution, and γ-CD changes the surrounding environment of the complex and shields the influence of the surrounding environment on the complex makes its structure more stable and reduces the energy loss in the process of electronic transition.

Fluorescence lifetime and quantum yield are important parameters to characterize fluorescent nanoparticles (see Supporting Information for calculation equations). The very long fluorescence lifetimes of lanthanide complexes, compared to the fluorescence lifetimes of organic fluorophores may also be used to distinguish signals and to enhance the signal relative to background autofluorescence in biological samples. The fluorescence lifetime of Eu(TTA)_3_phen complex and ECA with the highest fluorescence intensity in Figure 4b are shown in Figure 4c. The fluorescence lifetimes were 533.36 µs (complex) and 712.35 µs (ECA). The quantum yields were 53.8% (complex) and 62.9% (ECA). The fluorescence lifetime of the Tb(acac)_2_phen complex and TCA with the highest fluorescence intensity in Figure 4e are shown in Figure 4f. The fluorescence lifetimes were 135.70 µs (complex) and 233.35 µs (TCA). The quantum yields were 43.8% (complex) and 56.9% (TCA). From Figure 4c,f, it can be seen that the Eu^3+^ and Tb^3+^ complexes had increased fluorescence lifetime and quantum yield after being combined with γ-CD. The resulting ECA and TCA had the advantages of high fluorescence intensity, wide emission band, and long fluorescence lifetime.

### 3.3. Smart Sensing Property of Lanthanide (Eu^3+^ and Tb^3+^)-Loaded γ-Cyclodextrin Nano-Aggregates for Anticancer Drug of Irinotecan (CPT-11)

**Figure 5:**
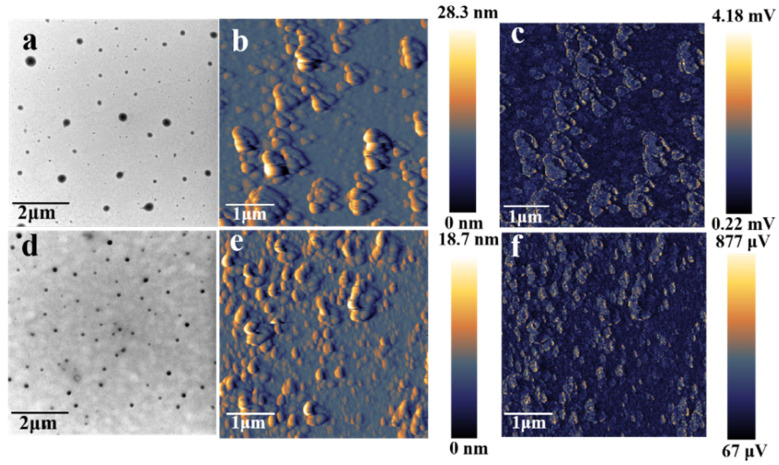
(**a**) TEM image of ECA-CPT-11. (**b**) Atomic force micrograph of ECA-CPT-11. (**c**) Photo-induced force micrograph of ECA-CPT-11. (**d**) TEM image of TCA-CPT-11. (**e**) Atomic force micrograph of TCA-CPT-11. (**f**) Photo-induced force micrograph of TCA-CPT-11.

The Eu^3+^ and Tb^3+^-loaded γ-cyclodextrin nano-aggregates have outstanding advantages, such as water solubility, nontoxicity, sharp emission, and long fluorescence lifetimes. Thus, the feasibility of these lanthanide-loaded γ-cyclodextrin nano-aggregates in developing highly-sensitive sensors for anticancer drugs was evaluated. First, TEM was used to evaluate the structures of ECA and TCA in the presence of drugs. Figure 5a and Figure 5d, respectively, show the TEM images of ECA and TCA combined with CPT-11 at a concentration of 100 μM. The sizes of the two nanoparticles became larger after the addition of the drug, and the size of ECA combined with CPT-11 was still larger than that of TCA. This demonstrates that the drug is interacting with the nanoparticles to form larger agglomerates. In addition, ECA and TCA samples combined to CPT-11 were imaged using photo-induced force microscopy (PiFM). PiFM excites the infrared absorption of a polarizable dipole in the sample, and detects this excitation locally by the force induced on the metal-coated atomic force microscope tip. Both excitation and detection of the sample occur in the near field, enabling high-resolution mapping of the absorption in the sample. This provides nanometer resolution of material morphology (via atomic force microscope) and composition distribution (via wavelength-dependent excitations). Figure 5b,c shows the atomic force microscope and PiFM images of ECA combined with CPT-11. The atomic force micrograph in Figure 5b shows the morphology of the aggregates after combining ECA with CPT-11. The morphology was slightly different from that seen in the TEM image in Figure 5a. The spin coating used to prepare the samples for AFM produces a centrifugal force on the sample, which changed the sample morphology. Consistent with this interpretation, it can be seen from Figure 5b that the morphology changes were oriented in a particular direction. Figure 5c is a PiFM image of the C-N single bond at 1083 cm^−1^, showing the distribution of CPT-11 in ECA aggregates. Figure 5e,f shows the atomic force microscope and PiFM images of TCA combined with CPT-11. The changes in morphology and the distribution of CPT-11 in TCA aggregates were consistent with the results of ECA combined with CPT-11. Comparing Figure 5b and Figure 5e, the size of ECA combined with CPT-11 under the atomic force microscope was still larger than that of TCA, which is consistent with the comparison of the shape and size under the TEM (Figure 5a,d).

**Figure 6:**
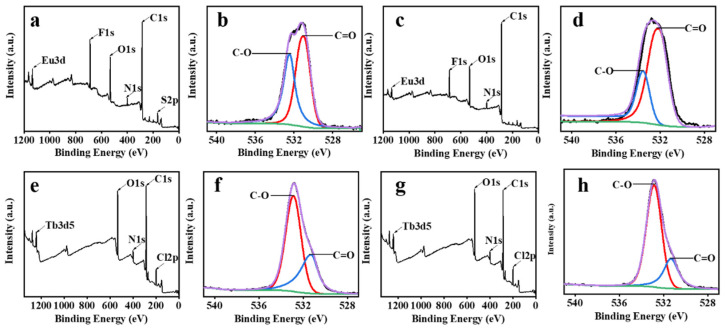
(**a**) XPS survey spectrum of ECA. (**b**) High-resolution O1s spectrum of ECA. (**c**) XPS survey spectrum of ECA-CPT-11. (**d**) High-resolution O1s spectrum of ECA-CPT-11. (**e**) XPS survey spectrum of TCA. (**f**) High-resolution O1s spectrum of TCA. (**g**) XPS survey spectrum of TCA-CPT-11. (**h**) High-resolution O1s spectrum of TCA-CPT-11.

X-ray photoelectron spectroscopy was also used to confirm the encapsulation of Eu^3+^ and Tb^3+^ complexes by γ-CD and the binding ability of ECA and TCA to CPT-11. Figure 6a, Figure 6c, Figure 6e and Figure 6g, respectively, show the XPS survey spectra of ECA, ECA loaded with CPT-11, TCA, and TCA loaded with CPT-11. The high-resolution O1s spectra of ECA and TCA are shown in Figure 6b and Figure 6f, respectively. Figure 6d and Figure 6h show the high-resolution O1s spectra of ECA and TCA loaded with CPT-11, respectively. As shown in Figure 6b, the O1s spectrum of ECA has two peaks, corresponding to C=O (531.01 eV) of ECA ligand TTA and C–O (532.43 eV) of γ-CD in ECA. The binding energies of C–O and C=O in ECA after adding γ-CD were increased by 1.05 eV and 0.33 eV, respectively, compared with Figure S4d and Figure 6b, indicating that O in γ-CD complexed with Eu^3+^ and changed the surrounding environment of Eu^3+^ complex, leading to an increase in the binding energy. Therefore, it was confirmed that γ-CD interacted with Eu^3+^ and complex to form ECA. Figure 6d shows that the high-resolution O1s spectrum of ECA loaded with CPT-11 had two peaks at 532.12 eV and 533.54 eV. Compared with Figure 6b, the carbon-oxygen binding energy in Figure 6d was increased. The increase of O1s binding energy may be due to the interaction between CPT-11 and ECA, which changed the local electronic structure and increased the binding energy. This indicates that CPT-11 entered the ECA nano aggregates. Similarly for TCA, as shown in Figure 6f, the O1s spectrum of TCA has two peaks, corresponding to C=O (531.24 eV) of TCA ligand acac and C–O (532.8 eV) of γ-CD in TCA. Compared with Figure S4h and Figure 6f, the binding energies of C–O and C=O were also increased in TCA. It was confirmed that γ-CD interacted with Tb^3+^ complex to form TCA. The high-resolution O1s spectrum after loading CPT-11 (Figure 6h) showed two peaks at 531.33 eV and 532.84 eV. Compared with Figure 6f, the binding energy of O1s in Figure 6h changes, indicating that CPT-11 interacts with TCA. This indicates that irinotecan entered the TCA nano aggregates.

**Figure 7:**
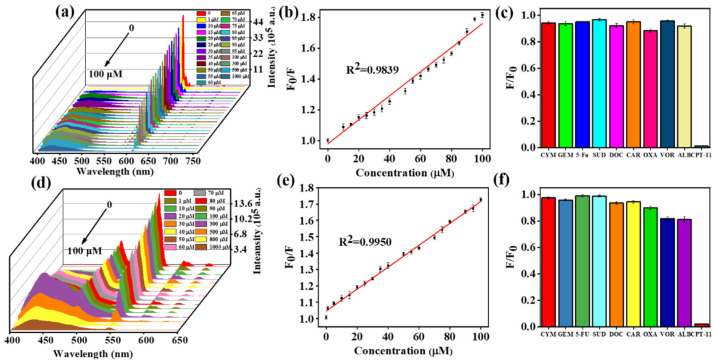
(**a**) Fluorescence emission spectra of ECA aggregates in the presence of different concentrations of CPT-11. (**b**) Fitting of Eu^3+^ quenching in the presence of CPT-11. (**c**) The degree of quenching of ECA by different drugs at 1 mM. (**d**) Fluorescence emission spectra of TCA aggregates in the presence of different concentrations of CPT-11. (**e**) Fitting of Tb^3+^ quenching in the presence of CPT-11. (**f**) The degree of quenching of TCA by different drugs at 1 mM. Error bars in e and f represent the standard deviation of *n* = 3 replicates for each condition.

Fluorescence spectroscopy was used to evaluate the fluorescence signal response of ECA and TCA to CPT-11. The sensitivity of ECA and TCA to different concentrations of CPT-11 was determined. Figure 7 shows the fluorescence emission spectra of ECA and TCA in the presence of different concentrations of CPT-11. The fluorescence emission spectra were obtained at the excitation wavelength of Eu^3+^ at λ_ex_ = 382 nm, and the excitation wavelength of Tb^3+^ at λ_ex_ = 334 nm, the maximum emission peak of Eu^3+^ at λ_em_ = 612 nm, and the maximum emission peak of Tb^3+^ at λ_em_ = 546 nm. As shown in Figure 7a,c, with the increase of CPT-11 concentration, the fluorescence signal intensity of ECA and TCA shows a gradual decrease. When the concentration of CPT-11 was 100 μM, the fluorescence intensity decreased by more than 50%. To further study the quenching efficiency of ECA and TCA fluorescence sensors, linear Stern–Volmer Equation (1) was used in the concentration ranges of 0–100 µM.

(1) $$\frac{F_{0}}{F}=1+K_{sv}\left[Q\right]$$

where *F*_0_ and *F* are, respectively, the fluorescence intensity of ECA and TCA at 612 nm and 546 nm in the absence and presence of CPT-11, *K_sv_* is the Stern–Volmer quenching constant, and [*Q*] is the drug concentration. As shown in Figure 7b,d, there was a good correlation coefficient between the quenching efficiency (*F*_0_/*F*) and the concentration of CPT-11 for ECA and TCA within 1–100 µM (R^2^ = 0.9839, 0.9950). The obtained linear Equations (2) and (3) were, respectively:

(2) $$\frac{F_{0}}{F}=0.9814+0.0078\left[Q\right]$$

(3) $$\frac{F_{0}}{F}=1.0459+0.0067\left[Q\right]$$

Within the concentration range of 0–100 μM, the limit of detection (LOD) of ECA and TCA for CPT-11 was calculated for $\frac{3\sigma }{S}$ (signal-to-noise ratio of 3) at 6.80 μM and 2.89 μM, respectively, where σ represents 20 blank measurements. The standard deviation of the value, *S* is the slope of the fitted curve.

The fluorescence-based detection of CPT-11 might be confounded by the presence of other drugs. Therefore, to detect the ability of ECA and TCA to *selectively* detect CPT-11, the fluorescence intensity of ECA and TCA was evaluated in the presence of nine other drugs: cyclophosphamide monohydrate (CYM), gemcitabine (GEM), 5-fluorouracil (5-Fu), sulfadiazine (SUD), Docetaxel (DOC), carvacrol (CAR), oxaliplatin (OXA), vorinostat (VOR) and albendazole (ALB), each at a concentration of 1 mM. Under the same test conditions and environment, the quenching effect of each anticancer drug on ECA and TCA is shown in Figure 7c,f. The relative fluorescence intensity, F/F_0_, value of CPT-11 was much lower than other anticancer drugs, demonstrating the high selectivity that ECA and TCA have for CPT-11.

### 3.4. Cytocompatibility of ECA and TCA

**Figure 8:**
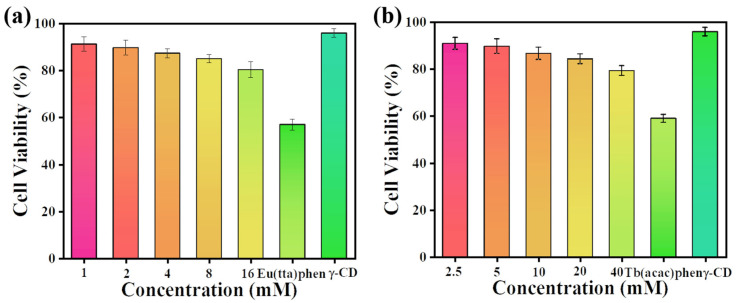
(**a**) Cell metabolic activity after incubation with Eu(TTA)_3_phen, γ-CD, and different concentrations of ECA for 24 h. (**b**) Cell metabolic activity after incubation with Tb(acac)_2_phen, γ-CD, and different concentrations of TCA for 24 h. Error bars represent standard deviation from *n* = 5 biological replicates for each condition.

The MTT method can be used to quantify cell viability [54]. Figure 8 shows the MTT cytotoxicity assays for Eu(TTA)_3_phen, Tb(acac)_2_phen, γ-CD, and five concentrations of ECA and TCA. Toxicity is defined here as a 20% reduction in the metabolic activity of cells measured by the MTT method. Eu(TTA)_3_phen (2 Mm) and Tb(acac)_2_phen (20 mM) complexes reduced the metabolic activity of HepG2 cells by about 40%, which meets our cytotoxicity threshold. As expected, γ-CD was not cytotoxic (>90% cell viability). Figure 8a,b shows that ECA and TCA at the five concentrations tested were not cytotoxic (>80% cell viability). In both cases, the presence of γ-CD reduced the cytotoxicity of the lanthanide complexes. A concentration of ECA eight times the tested cytotoxic concentration of Eu(TTA)_3_phen was non-cytotoxic, and a concentration of TCA two times higher than a cytotoxic concentration of Tb(acac)_2_phen was non-cytotoxic. These results confirmed our initial hypothesis that the inclusion of Eu(TTA)_3_phen and Tb(acac)_2_phen complexes by γ-CD would reduce the cytotoxicity of ECA and TCA, resulting in an excellent cytocompatibility.

### 3.5. Detection of CPT-11 in Cells

**Figure 9:**
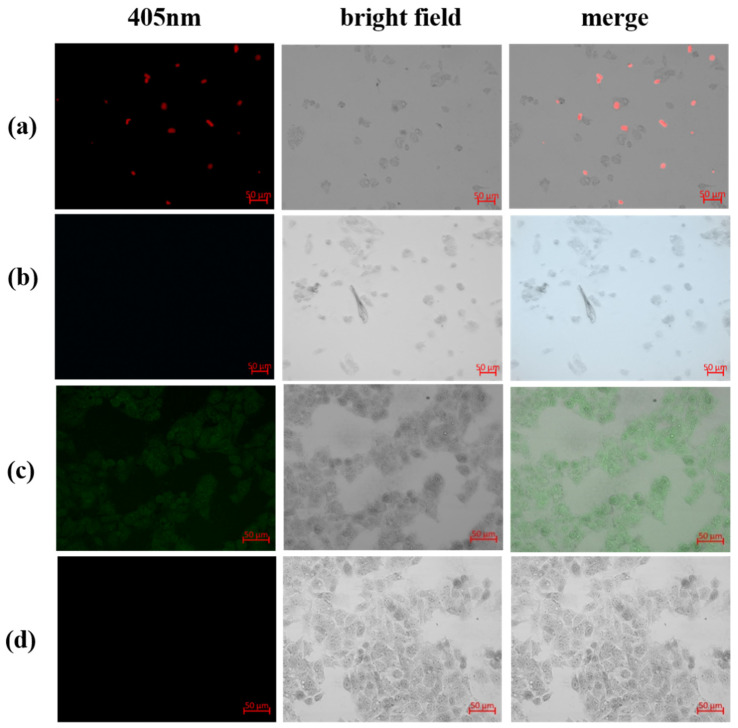
(**a**) Laser scanning confocal fluorescence image of HepG2 cells incubated with ECA nanoparticles and (**b**) ECA-containing cells after adding CPT-11. (**c**) Laser scanning confocal images of HepG2 cells incubated with TCA nanoparticles and, (**d**) TCA-containing cells after addition of CPT-11.

The low toxicity of ECA and TCA and the rapid response and high selectivity of CPT-11 towards ECA and TCA prompted us to further evaluate the sensing ability of this fluorescent sensor in living cells. Therefore, imaging of ECA and TCA in HepG2 cells was investigated. Figure 9 shows laser scanning confocal fluorescence images of HepG2 cells. After co-incubating ECA with HepG2 cells for 1 h, red fluorescence did not appear in the cells (Figure 9a), but red fluorescence continued to be observed outside the cells. The extracellular red fluorescence disappeared after the addition of CPT-11 solution (Figure 9b), indicating that CPT-11 could still quench the fluorescence of ECA in the cell culture medium. After co-incubating TCA with HepG2 cells for 1 h, obvious green fluorescence appeared in the cells (Figure 9c), which also indicated that TCA could effectively enter the cells. CPT-11 solution was added under the same conditions, and the fluorescence was quenched after TCA-containing cells were incubated with CPT-11 for 30 min (Figure 9d). This suggests that TCA could detect CPT-11 in cells. In summary, laser scanning confocal fluorescence images indicated that ECA and TCA could detect CPT-11 extracellularly and intracellularly, respectively.

## 4. Conclusions

In this article, we synthesized lanthanide (Eu^3+^ and Tb^3+^)-loaded γ-CD nano-aggregates, and the nano-aggregates enabled selective and sensitive detection of the anticancer drug CPT-11. The photophysical properties of these nano-aggregates included high fluorescence intensity, long fluorescence lifetime, and high quantum yield. These fluorescent nanoparticles could detect the concentration of CPT-11 in situ with cells and had sensitive selectivity for CPT-11. These fluorescent nano-aggregates were demonstrated to be nontoxic and cytocompatible by a cytotoxicity assay (MTT). Furthermore, we demonstrated that TCA nano-aggregates could label cells and detect intracellular CPT-11. In contrast, ECA nano-aggregates could detect CPT-11 in the extracellular environment without causing cell death. Therefore, ECA and TCA have the potential to serve as drug sensors with high fluorescence intensity, water stability, high sensitivity, and selectivity for in vivo sensing of the anticancer drug CPT-11.

## Figures and Tables

**Table 1:** Solution concentrations used to obtain ECA and TCA.

Sample	C_Eu_	C_Tb_	C_TTA_	C_acac_	C_Phen_	C_γ-CD_
	(mol/L)	(mol/L)	(mol/L)	(mol/L)	(mol/L)	(mol/L)
**A**	1.0 × 10^−4^	0	3.0 × 10^−4^	0	1.0 × 10^−4^	3.3 × 10^−4^
**B**	1.0 × 10^−3^	0	3.0 × 10^−3^	0	1.0 × 10^−3^	3.3 × 10^−3^
**C**	0	1.0 × 10^−3^	0	2.0 × 10^−3^	1.0 × 10^−3^	1.0 × 10^−4^
**D**	0	1.0 × 10^−2^	0	2.0 × 10^−2^	1.0 × 10^−2^	1.0 × 10^−3^

## Data Availability

Not applicable.

## Supplementary Materials

The following supporting information can be downloaded at: https://www.mdpi.com/article/10.3390/ijms23126597/s1.

## References

[1] Vogeser M. (2020). From therapeutic drug monitoring to total drug monitoring and drug-omics. Clin. Chem. Lab. Med..

[2] Luan F., He X., Zeng N. (2020). Tetrandrine: A review of its anticancer potentials, clinical settings, pharmacokinetics and drug delivery systems. J. Pharm. Pharmacol..

[3] Krens S.D., Lassche G., Jansman F.G.A., Desar I.M.E., Lankheet N.A.G., Burger D.M., Van Herpen C.M.L., Van Erp N.P. (2019). Dose recommendations for anticancer drugs in patients with renal or hepatic impairment. Lancet Oncol..

[4] Liu J., Sun L., Li L., Zhang R., Xu Z.P. (2021). Synergistic cancer photochemotherapy via layered double hydroxide-based trimodal nanomedicine at very low therapeutic doses. ACS Appl. Mater. Interfac..

[5] Huang W., Chen L., Kang L., Jin M., Sun P., Xin X., Gao Z., Bae Y.H. (2017). Nanomedicine-based combination anticancer therapy between nucleic acids and small-molecular drugs. Adv. Drug Deliv. Rev..

[6] Alvau M.D., Tartaggia S., Meneghello A., Casetta B., Calia G., Serra P.A., Polo F., Toffoli G. (2018). Enzyme-based electrochemical biosensor for therapeutic drug monitoring of anticancer drug irinotecan. Anal. Chem..

[7] Lavezzi S.M., Borella E., Carrara L., De Nicolao G., Magni P., Poggesi I. (2018). Mathematical modeling of efficacy and safety for anticancer drugs clinical development. Expert Opin. Drug Discov..

[8] Wu X., Liu Q., Zhang K., Cheng M., Xin X. (2018). Optimal switching control for drug therapy process in cancer chemotherapy. Eur. J. Control..

[9] Komen J., Westerbeek E.Y., Kolkman R.W., Roesthuis J., Lievens C., Van den Berg A., Van der Meer A.D. (2020). Controlled pharmacokinetic anti-cancer drug concentration profiles lead to growth inhibition of colorectal cancer cells in a microfluidic device. Lab Chip.

[10] Shindi O., Kanesan J., Kendall G., Ramanathan A. (2020). The combined effect of optimal control and swarm intelligence on optimization of cancer chemotherapy. Comput. Methods Programs Biomed..

[11] Hifumi T., Miyoshi N., Kawaguchi H., Nomura K., Yasuda N. (2010). Immunohistochemical detection of proteins associated with multidrug resistance to anti-cancer drugs in canine and feline primary pulmonary carcinoma. J. Vet. Med. Sci..

[12] Wu J., Crist R.M., McNeil S.E., Clogston J.D. (2019). Ion quantification in liposomal drug products using high performance liquid chromatography. J. Pharm. Biomed. Anal..

[13] Safaei M., Shishehbore M.R. (2021). A review on analytical methods with special reference to electroanalytical methods for the determination of some anticancer drugs in pharmaceutical and biological samples. Talanta.

[14] Brothman A.R., Davis T.P., Duffy J.J., Lindell T.J. (1982). Development of an Antibody to Actinomycin D and Its Application for the Detection of Serum Levels by Radioimmunoassay. Cancer Res..

[15] Mullapudi S.S., Mitra D., Li M., Kang E.-T., Chiong E., Neoh K.G. (2020). Potentiating anti-cancer chemotherapeutics and antimicrobials via sugar-mediated strategies. Mol. Syst. Des. Eng..

[16] Meisenberg C., Ashour M.E., El-Shafie L., Liao C., Hodgson A., Pilborough A., Khurram S.A., Downs J.A., Ward S.E., El-Khamisy S.F. (2017). Epigenetic changes in histone acetylation underpin resistance to the topoisomerase I inhibitor irinotecan. Nucleic Acids Res..

[17] Bolat G. (2020). Investigation of poly (CTAB-MWCNTs) composite based electrochemical DNA biosensor and interaction study with anticancer drug Irinotecan. Microchem. J..

[18] Yamaguchi T., Iwasa S., Shoji H., Honma Y., Takashima A., Kato K., Hamaguchi T., Higuchi K., Boku N. (2019). Association between UGT1A1 gene polymorphism and safety and efficacy of irinotecan monotherapy as the third-line treatment for advanced gastric cancer. Gastric Cancer.

[19] Gold H.T., Hall M.J., Blinder V., Schackman B.R. (2009). Cost effectiveness of pharmacogenetic testing for uridine diphosphate glucuronosyltransferase 1A1 before irinotecan administration for metastatic colorectal cancer. Cancer.

[20] Tsai H.L., Huang C.W., Lin Y.W., Wang J.H., Wu C.C., Sung Y.C., Chen T.L., Wang H.M., Tang H.C., Chen J.B. (2020). Determination of the UGT1A1 polymorphism as guidance for irinotecan dose escalation in metastatic colorectal cancer treated with first-line bevacizumab and FOLFIRI (PURE FIST). Eur. J. Cancer.

[21] De Man F.M., Goey A.K.L., Van Schaik R.H.N., Mathijssen R.H.J., Bins S. (2018). Individualization of irinotecan treatment: A review of pharmacokinetics, pharmacodynamics, and pharmacogenetics. Clin. Pharmacokinet..

[22] Allegrini G., Falcone A., Fioravanti A., Barletta M.T., Orlandi P., Loupakis F., Cerri E., Masi G., Di Paolo A., Kerbel R.S. (2008). A pharmacokinetic and pharmacodynamic study on metronomic irinotecan in metastatic colorectal cancer patients. Br. J. Cancer.

[23] Zhuang Q., Liu X., Sun Z., Wang H., Jiang J. (2019). A validated UPLC-MS/MS method to determine free and total irinotecan and its two metabolites in human plasma after intravenous administration of irinotecan hydrochloride liposome injection. J. Pharm. Biomed. Anal..

[24] Puscasu A., Zanchetta M., Posocco B., Bunka D., Tartaggia S., Toffoli G. (2021). Development and validation of a selective SPR aptasensor for the detection of anticancer drug irinotecan in human plasma samples. Anal. Bioanal. Chem..

[25] Marangon E., Posocco B., Mazzega E., Toffoli G. (2015). Development and validation of a high-performance liquid chromatography-tandem mass spectrometry method for the simultaneous determination of irinotecan and its main metabolites in human plasma and its application in a clinical pharmacokinetic study. PLoS ONE.

[26] Bonazza G., Tartaggia S., Toffoli G., Polo F., Daniele S. (2018). Voltammetric behaviour of the anticancer drug irinotecan and its metabolites in acetonitrile. Implications for electrochemical therapeutic drug monitoring. Electrochim. Acta.

[27] Aleem A.R., Ding W., Liu J., Li T., Guo Y., Wang Q., Wang Y., Wang Y., Rehman F.U.L., Kipper M.J. (2021). Visible-light excitable Eu(3+)-induced hyaluronic acid-chitosan aggregates with heterocyclic ligands for sensitive and fast recognition of hazardous ions. Int. J. Biol. Macromol..

[28] Wang Z., Qiu X., Xi W., Tang M., Liu J., Jiang H., Sun L. (2021). Tailored upconversion nanomaterial: A hybrid nano fluorescent sensor for evaluating efficacy of lactate dehydrogenase inhibitors as anticancer drugs. Sens. Actuators B Chem..

[29] Aleem A.R., Liu J., Wang J., Wang J., Zhao Y., Wang Y., Wang Y., Wang W., Rehman F.U., Kipper M.J. (2020). Selective sensing of Cu(2+) and Fe(3+) ions with vis-excitation using fluorescent Eu(3+)-Induced aggregates of polysaccharides (EIAP) in mammalian cells and aqueous systems. J. Hazard. Mater..

[30] Song Z., Wang J., Wang J., Liu J., Wang X., Wang Y., Aleem A.R., Kipper M.J., Belfiore L.A., Tang J. (2021). Eu3+-induced polysaccharide nano-dumbbell aggregates (PNDA) as drug carriers to smartly report drug concentration through variable fluorescence. Sens. Actuators B Chem..

[31] Wang J., Liu J., Wang J., Wang Y., Cao J., Hou L., Ge R., Chi J., Huang L., Guo J. (2020). Smart sensing of Cu2+ in living cells by water-soluble and nontoxic Tb3+/Eu3+-induced aggregates of polysaccharides through fluorescence imaging. J. Mater. Chem. C.

[32] Parveen S., Prasanna P.K., Chakraborty S., Giri P.K. (2021). Stable deep blue emission with unity quantum yield in organic–inorganic halide perovskite 2D nanosheets doped with cerium and terbium at high concentrations. J. Mater. Chem. C.

[33] Zhao Z., Bian M., Lin C., Fu X., Yu G., Wei H., Liu Z., Bian Z., Huang C. (2021). Efficient green OLEDs achieved by a terbium (III) complex with photoluminescent quantum yield close to 100%. Sci. China Chem..

[34] Su B., Yang W., Wang Y., Huang L., Popat K.C., Kipper M.J., Belfiore L.A., Tang J. (2020). Europium-functionalized luminescent titania nanotube arrays: Utilizing interactions with glucose, cholesterol and triglycerides for rapid detection application. Mater. Sci Eng. C Mater. Biol. Appl..

[35] Cernea M., Secu M., Radu R., Ganea P., Surdu V.A., Trusca R., Vasile E.T., Secu E.C. (2021). Structural, electrical properties and photoluminescence analyses of the terbium doped barium titanate. J. Alloys Compd..

[36] Wang J., Wang J., Liu J., Wang X., Aleem A.R., Song Z., Kipper M.J., Tang J. (2020). Smart sensing of bacterial contamination on fluorescent cotton fabrics (FCF) by nontoxic Eu3+-induced polyelectrolyte nano-aggregates (EIPAs). Dye. Pigment..

[37] Rong M., Ye J., Chen B., Wen Y., Deng X., Liu Z.-Q. (2020). Ratiometric fluorescence detection of stringent ppGpp using Eu-MoS2 QDs test paper. Sens. Actuators B Chem..

[38] Xu Q., Li Z., Li H. (2016). Water-soluble luminescent hybrid composites consisting of oligosilsesquioxanes and lanthanide complexes and their sensing ability for Cu2+. Chem.—A Eur. J..

[39] Morin-Crini N., Fourmentin S., Fenyvesi É., Lichtfouse E., Torri G., Fourmentin M., Crini G. (2021). 130 years of cyclodextrin discovery for health, food, agriculture, and the industry: A review. Environ. Chem. Lett..

[40] Votava M., Ravoo B.J. (2021). Principles and applications of cyclodextrin liquid crystals. Chem Soc. Rev..

[41] Crini G. (2014). Review: A history of cyclodextrins. Chem. Rev..

[42] Wang L., Xia Y., Su L., Wu J. (2020). Modification of bacillus clarkii gamma-cyclodextrin glycosyltransferase and addition of complexing agents to increase gamma-cyclodextrin production. J. Agric. Food Chem.

[43] Gattuso G., Nepogodiev S.A., Stoddart J.F. (1998). Synthetic cyclic oligosaccharides. Chem. Rev..

[44] Roy I., Stoddart J.F. (2021). Cyclodextrin metal-organic frameworks and their applications. Acc. Chem. Res..

[45] Dodero A., Schlatter G., Hebraud A., Vicini S., Castellano M. (2021). Polymer-free cyclodextrin and natural polymer-cyclodextrin electrospun nanofibers: A comprehensive review on current applications and future perspectives. Carbohydr. Polym..

[46] Fan W., An W., Huo M., Xiao D., Lyu T., Cui J. (2021). An integrated approach using ozone nanobubble and cyclodextrin inclusion complexation to enhance the removal of micropollutants. Water Res..

[47] Somsri S., Kuwamura N., Kojima T., Yoshinari N., Konno T. (2020). Self-assembly of cyclic hexamers of gamma-cyclodextrin in a metallosupramolecular framework with d-penicillamine. Chem. Sci..

[48] Majd M., Yazdanpanah M., Bayatloo M.R., Nojavan S. (2021). Recent advances and applications of cyclodextrins in magnetic solid phase extraction. Talanta.

[49] Dos Santos Lima B., Shanmugam S., De Souza Siqueira Quintans J., Quintans-Júnior L.J., De Souza Araújo A.A. (2019). Inclusion complex with cyclodextrins enhances the bioavailability of flavonoid compounds: A systematic review. Phytochem. Rev..

[50] Xiao Z., Zhang Y., Niu Y., Ke Q., Kou X. (2021). Cyclodextrins as carriers for volatile aroma compounds: A review. Carbohydr. Polym..

[51] Noël S., Léger B., Ponchel A., Sadjadi S., Monflier E. (2021). Cyclodextrins as multitask agents for metal nano-heterogeneous catalysis: A review. Environ. Chem. Lett..

[52] Zhang E., Xing R., Liu S., Li K., Qin Y., Yu H., Li P. (2019). Vascular targeted chitosan-derived nanoparticles as docetaxel carriers for gastric cancer therapy. Int. J. Biol. Macromol..

[53] Li Y., Kröger M., Liu W.K. (2015). Shape effect in cellular uptake of PEGylated nanoparticles: Comparison between sphere, rod, cube and disk. Nanoscale.

[54] Pintor A.V.B., Queiroz L.D., Barcelos R., Primo L.S.G., Maia L.C., Alves G.G. (2020). MTT versus other cell viability assays to evaluate the biocompatibility of root canal filling materials: A systematic review. Int. Endod. J..

