# Supplementary material for: Lanthanide (Eu3+/Tb3+)-Loaded γ-Cyclodextrin Nano-Aggregates for Smart Sensing of the Anticancer Drug Irinotecan
Source: Int J Mol Sci. 2022 Jun 13;23(12):6597. doi: 10.3390/ijms23126597 (PMC9223530; doi:10.3390/ijms23126597)
Supplement: Supplementary file 1 [file ijms-23-06597-s001.zip › ijms-1769460-supplementary.pdf]

International Journal of *Molecular Sciences*

MDPI

Lanthanide (Eu^{3+}/Tb^{3+})-Loaded γ-Cyclodextrin Nano-Aggregates for Smart Sensing of the Anticancer Drug Irinotecan

Yaowei Guo [1], Jin Liu [1], Qinglin Tang [1], Cuicui Li [1], Yanying Zhang [1], Yao Wang [1], Yanxin Wang [1], Yupeng Bi [1], Christopher D. Snow [2], Matt J. Kipper [2,*], Laurence A. Belfiore [2] and Jianguo Tang [1,*]

1 Institute of Hybrid Materials, National Center of International Joint Research for Hybrid Materials Technology, National Base of International Sci. & Tech. Cooperation on Hybrid Materials, Qingdao University, 308 Ningxia Road, Qingdao 266071, China; yw1996guo@163.com (Y.G.); liujin0620@126.com (J.L.); a15666920912@163.com (Q.T.); 17853482427@163.com (C.L.); zyyaaaa@163.com (Y.Z.); wangyaoqdu@126.com (Y.W.); wangyanxin@qdu.edu.cn (Y.W.); b15254156675@163.com (Y.B.)

2 Department of Chemical and Biological Engineering, Colorado State University, Fort Collins, CO 80523, USA; christopher.snow@colostate.edu (C.D.S.); belfiore@engr.colostate.edu (L.A.B.)

* Correspondence: matthew.kipper@colostate.edu (M.J.K.); jianguotangde@hotmail.com (J.T.); Tel.: +86-532-85952561 (J.T.); Fax: +86-532-85951519 (J.T.)

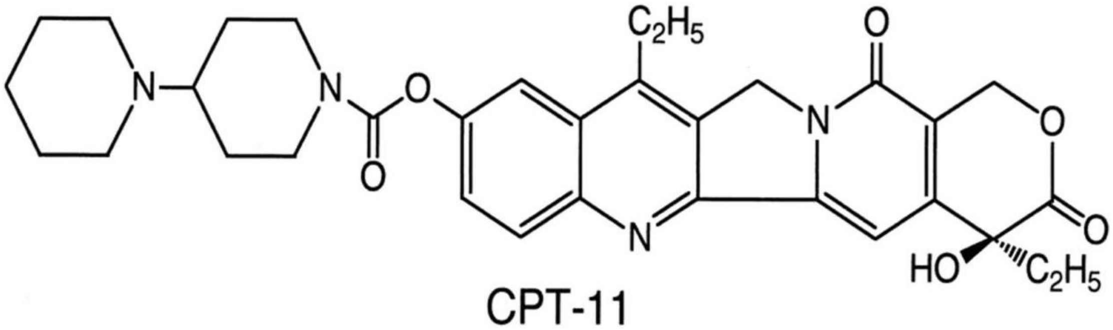

Figure S1. Chemical structure of irinotecan [1].

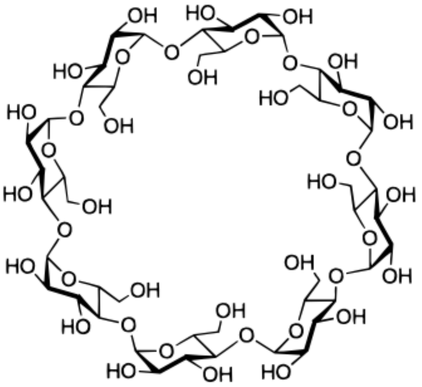

Figure S2. Chemical structure of γ-cyclodextrin [2].

Fluorescence lifetime and quantum yield

Fluorescence lifetime (τ_{obs}) and quantum yield (Φ_{tot}) measurements were recorded by using the Edinburg instrument (FLS 1000) Edinburgh, UK.

The fluorescence lifetime decay curve of samples was measured by μF2 lamp model with 100 W power and 90-260 V supply. All the decay curves can be fitted well by a decay Eq. described as

$$I(t) = A + B_1 \exp(-t/\tau_1) + B_2 \exp(-t/\tau_2) \quad \text{Eq.1.1} \quad (1)$$

Where I is the fluorescence intensity at time t, τ_1 and τ_2 are the decay times and parameter A, B_1, and B_2 is the fitting constant.

The fluorescence quantum yield (Φ_{tot}) is related to the number of photons absorbed (α) and the number of photons emitted by the sample (ε). described as

$$\Phi = \frac{\varepsilon}{\alpha} = \frac{\int I_{emission}}{\int I_{solvent} - \int I_{sample}} \quad \text{Eq.1.2} \qquad (2)$$

where $I_{emission}$ is luminescence emission spectrum of sample, $I_{solvent}$ is the spectrum of light used to excite only solvent, and I_{sample} is the spectrum of light used for exciting sample in solvent. All spectra are collected using the integrating sphere.

(a)

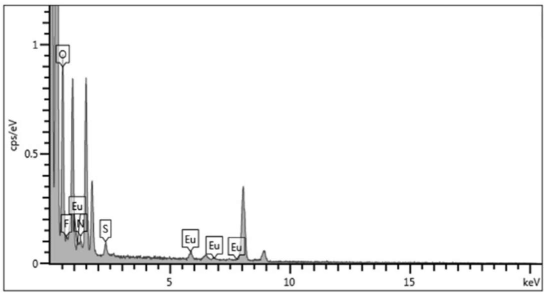

(b)

Element	Weight (%)
O	59.85
Eu	12.48
F	19.05
N	5.97
S	2.65

(c)

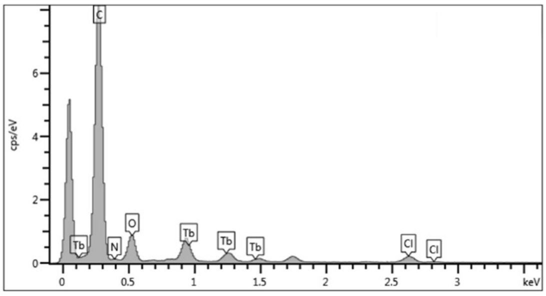

(d)

Element	Weight (%)
C	64.32
Tb	4.45
N	2.75
O	28.17
Cl	1.21

Figure S3. (**a**) and (**b**) relative contents of elements in sample B tested by EDS, (c) and (d) relative contents of elements in sample D tested by EDS.

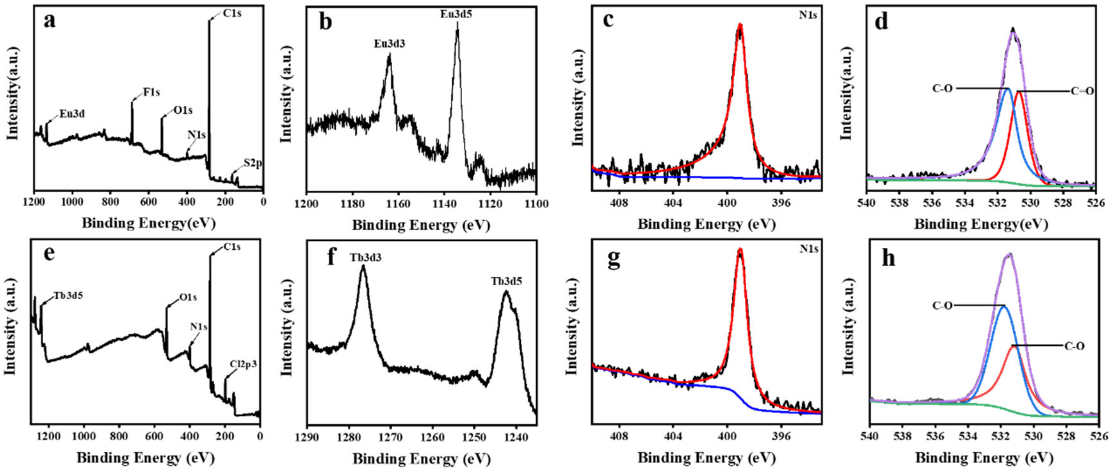

Figure S4. (a) XPS survey spectrum of Eu^{3+}complex. (b) High resolution Eu3d spectrum of Eu^{3+}complex. (c) High resolution N1s spectrum of Eu^{3+}complex. (d) High resolution O1s spectrum of Eu^{3+}complex. (e) XPS survey spectrum of Tb^{3+} complex. (f) High resolution Tb3d spectrum of Tb^{3+} complex. (g) High resolution N1s spectrum of Tb^{3+}complex. (h) High resolution O1s spectrum of Tb^{3+}complex.

References

1. Bolat, G., Investigation of poly(CTAB-MWCNTs) composite based electrochemical DNA biosensor and interaction study with anticancer drug Irinotecan. *Microchemical Journal* **2020,** 159, 105426.
2. Gattuso, G.; Nepogodiev, S. A.; Stoddart, J. F., Synthetic Cyclic Oligosaccharides. *Chemical Reviews* **1998,** 98, (5), 1919-1958.
